# Local bifurcation with spin-transfer torque in superparamagnetic tunnel junctions

**DOI:** 10.1038/s41467-022-31788-1

**Published:** 2022-07-14

**Authors:** Takuya Funatsu, Shun Kanai, Jun’ichi Ieda, Shunsuke Fukami, Hideo Ohno

**Affiliations:** 1 Laboratory for Nanoelectronics and Spintronics, Research Institute of Electrical Communication, Tohoku University, Sendai, Japan; 2 PRESTO, Japan Science and Technology Agency, Kawaguchi, Japan; 3 Division for the Establishment of Frontier Sciences, Tohoku University, Sendai, Japan; 4 Center for Science and Innovation in Spintronics, Tohoku University, Sendai, Japan; 5 Advanced Science Research Center, Japan Atomic Energy Agency, Tokai, Japan; 6 WPI-Advanced Institute for Materials Research, Tohoku University, Sendai, Japan; 7 Center for Innovative Integrated Electronic Systems, Tohoku University, Sendai, Japan; 8 Inamori Research Institute for Science, Kyoto, Japan

**Keywords:** Electrical and electronic engineering, Phase transitions and critical phenomena, Magnetic devices

## Abstract

Modulation of the energy landscape by external perturbations governs various thermally-activated phenomena, described by the Arrhenius law. Thermal fluctuation of nanoscale magnetic tunnel junctions with spin-transfer torque (STT) shows promise for unconventional computing, whereas its rigorous representation, based on the Néel-Arrhenius law, has been controversial. In particular, the exponents for thermally-activated switching rate therein, have been inaccessible with conventional thermally-stable nanomagnets with decade-long retention time. Here we approach the Néel-Arrhenius law with STT utilising superparamagnetic tunnel junctions that have high sensitivity to external perturbations and determine the exponents through several independent measurements including homodyne-detected ferromagnetic resonance, nanosecond STT switching, and random telegraph noise. Furthermore, we show that the results are comprehensively described by a concept of local bifurcation observed in various physical systems. The findings demonstrate the capability of superparamagnetic tunnel junction as a useful tester for statistical physics as well as sophisticated engineering of probabilistic computing hardware with a rigorous mathematical foundation.

## Abstract (web summary)

There has been much interest in using the probabilistic switching of magnetic tunnel junctions in unconventional computing, but to do so requires a detailed understanding of this switching. Here, Funatsu et al rigorously determine the switching exponents in superparamagnetic tunnel junctions.

## Introduction

A dynamical system is classified by the stability of its potential landscape, especially by the local bifurcation of the dynamical equation. Under finite stochasticity, the Arrhenius law, a general principle for thermally-activated events, describes a variety of dynamical phenomena ranging from chemical reactions to physical processes. According to the Arrhenius law, the relaxation time *τ* in staying at a certain state is given by *τ* = *τ*_0_ exp*Δ* with the thermal stability factor *Δ* ≡ *E*_0_/*k*_B_*T*, where *τ*_0_ is the intrinsic time constant of each system, *k*_B_ the Boltzmann constant, *T* the absolute temperature, and *E*_0_ an intrinsic energy barrier for switching to different states without perturbation. Under the perturbation by normalised external input *x*, *E*_0_ is replaced by an effective energy barrier *E* = ${E}_{0}{\left(1-x\right)}^{{n}_{x}}$, where the switching exponent ${n}_{x}$ is determined by the effective energy landscape with *x*. In general, when the dynamical equation d*Θ*/d*t* = *f*(*Θ*,*x*) is given, where *t* is the time and *Θ* is the state variable (e.g. particle position, spin quantisation direction, amount of unreacted substance, etc.), the types of local bifurcation of *f*(*Θ*,*x*) determines ${n}_{x}$; for example, ${n}_{x}$ = 2 for the pitchfork bifurcation and ${n}_{x}$ = 3/2 for the saddle-node bifurcation^1^. This suggests that the switching exponent serves as a unique lens for the local structure, especially the stability of the energy landscape under the perturbations in the relevant system.

In magnetic materials, which have served as a model system to study the physics of thermally-activated phenomena, the basis of the Arrhenius law was built by Néel^2^ and Brown^3^, known as the Néel–Arrhenius law. For single-domain uniaxial magnets with a magnetic field *H* applied along the easy axis, *E* under magnetic field can be simply derived as *E* = *E*_0_(1 – *H*/*H*_*K*_^eff^)^2^ by the Stoner–Wohlfarth model, where *H*_*K*_^eff^ is the effective magnetic anisotropy field^4^. However, theoretical studies pointed out that the value of exponent *n*_*H*_, 2 in the above equation, should vary when one considers some realistic factors such as misalignment of magnetic field^5,6^ and higher-order terms of anisotropy^7^; in other words, the local bifurcation varies with them.

The magnetisation of nanomagnets can also be controlled by spin-transfer torque (STT) under current application through the angular momentum transfer^8–13^. The STT-induced magnetisation switching in thermally-stable magnetic tunnel junctions (MTJ) is a key ingredient for non-volatile magnetoresistive random access memory^14–16^. Moreover, recent studies have demonstrated an unconventional paradigm of computing, e.g. neuromorphic computing with population coding^17^, and probabilistic computing^18^, which utilises a combinatorial effect of STT and thermal fluctuation in superparamagnetic tunnel junctions (s-MTJs). By further combining the effect of external magnetic fields, additional tunabilities of the s-MTJs for probabilistic computing have been shown^19^. In this regard, understanding how the effective energy of s-MTJs is characterised under STT, as well as magnetic field, is of significant interest not only from fundamental but also from technological aspects. The Néel–Arrhenius law under STT has been a long-standing question partly because the STT itself does not modulate the energy landscape due to its non-conservative nature, preventing one from defining its effective potential energy. Despite the difficulty, the expectation value of event time of the magnetisation switching, i.e. the Néel relaxation time *τ*, under field *H* and current *I* is phenomenologically expressed in a form:

1 $$\tau ={\tau }_{0}{{\exp }}\left[\frac{{E}_{0}}{{k}_{{{{{{\rm{B}}}}}}}T}{\left(1-\frac{H}{{H}_{K}^{{{{{{\rm{eff}}}}}}}}\right)}^{{n}_{H}}{\left(1-\frac{I}{{I}_{{{{{{\rm{C}}}}}}0}}\right)}^{{n}_{I}}\right],$$

where *τ*_0_ is ~1 ns in magnetic systems^3^, and *I*_C0_ an intrinsic critical current. Regarding the exponent *n*_*I*_ for the factor of current, different values, 1 (refs. ^20,21^) or 2 (refs. ^22–24^), have been theoretically derived, where the former was obtained by considering a fictitious temperature, whereas the latter was obtained by analysing the stochastic process based on the Fokker–Planck equation. Experimentally, it has been practically inaccessible as far as one examines conventional thermally-stable MTJs and consequently, their decade-long unperturbed retention property has been extrapolated from limited data obtained in a reasonable time while assuming a certain number for *n*_*H*_ or *n*_*I*_^25–33^. For applications with superparamagnetic tunnel junctions that actively utilise thermal fluctuation under STT, such uncertainty makes sophisticated engineering impractical as a rigorous description of modulation of the effective energy landscape is indispensable.

Here we experimentally study the Néel–Arrhenius law of a nanomagnet under STT utilising superparamagnetic tunnel junctions that allow direct determination of the event time under fields and currents^34–39^. Through measurements of homodyne-detected ferromagnetic resonance (FMR) under current biases, high-speed STT switching with various pulse widths, and random telegraph noise (RTN) under various fields and currents, values of *n*_*H*_ and *n*_*I*_ are uniquely determined for given conditions. Furthermore, we show that by considering the local bifurcations under magnetic field and STT, which have not been considered for magnetic systems, the obtained results can be comprehended with the effects of the torques of the field and current without the difficulties to define the effective potential of STT.

## Sample preparation and strategy of following experiments

**Fig. 1:**
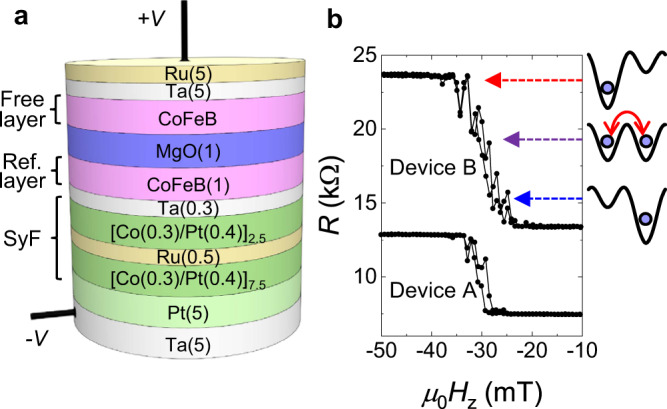
Sample structure and *R*–*H*_*z*_ property. **a** Stack structure of the magnetic tunnel junction. **b** Resistance *R* as a function of external perpendicular magnetic field *H*_*z*_.

As shown in Fig. 1a, a stack structure, Ta(5)/Pt(5)/[Co(0.3)/Pt(0.4)]_7_/Co(0.3)/Ru(0.45)/[Co(0.3)/Pt(0.4)]_2_/Co(0.3)/Ta(0.3)/CoFeB/MgO(1.0)/CoFeB(*t*_CoFeB_ = 1.88)/Ta(5)/Ru(5) (numbers in parenthesis are nominal thickness in nm), is deposited by dc/rf magnetron sputtering on a sapphire substrate. The stack possesses essentially the same structure as what was utilised in the demonstration of probabilistic computing^18^. Resistance (*R*)-area (*A*) product, *RA*, of the MgO tunnel barrier is 5.5 Ωμm^2^. The stack is patterned into MTJ devices, followed by annealing at 300 °C. The MTJ device we will mainly focus on hereafter (device A) has a diameter *D* of 34 nm (results for device B with *t*_CoFeB_ = 1.82 nm, *RA* = 8.1 Ωμm^2^, and *D* = 28 nm will be also shown later). In this size range, the magnetisation can be represented by a single vector without significant effects of spatial inhomogeneity for the present stack structure^16,27,40^. Both CoFeB layers have a perpendicular easy axis. Figure 1b shows the junction resistance *R* as a function of the perpendicular magnetic field *H*_*z*_. Gradual variation of mean *R* with *H*_*z*_ and scattering of data points at the transition region reflects a superparamagnetic nature of the MTJ whose switching time is shorter than the measurement time of *R* (~ 1 s).

To determine *n*_*H*_ and *n*_*I*_ in actual MTJs, we take into account the following two effects: electric-field modulation of magnetic anisotropy^41–44^ and uncompensated stray field *H*_S_ from the reference layer. Consequently, *Δ*, the argument of the exponential in Eq. (1), is rewritten as

2 $${\it\varDelta }_{{{{{{\rm{P}}}}}}({{{{{\rm{AP}}}}}})}	= \,\frac{{E}_{0}}{{k}_{{{{{{\rm{B}}}}}}}T}{\left(1\pm \frac{{H}_{z}-{H}_{{{{{{\rm{S}}}}}}}}{{H}_{K}^{{{{{{\rm{eff}}}}}}}(V)}\right)}^{{n}_{H}}{\left(1-\frac{V}{{V}_{{{{{{\rm{C}}}}}}0,{{{{{\rm{P}}}}}}({{{{{\rm{AP}}}}}})}}\right)}^{{n}_{I}}\\ \,	\equiv \,{\it\varDelta }_{0}{(1\pm h({H}_{z},V))}^{{n}_{H}}{(1-{v}_{{{{{{\rm{P}}}}}}({{{{{\rm{AP}}}}}})}(V))}^{{n}_{I}},$$

with ${{\it\Delta}}_{0}\equiv {E}_{0}/{k}_{{{{{{\rm{B}}}}}}}T$, $h({H}_{z},V)\equiv \left({H}_{z}-{H}_{{{{{{\rm{S}}}}}}}\right)/{H}_{K}^{{{{{{\rm{eff}}}}}}}(V)$, and ${v}_{{{{{{\rm{P}}}}}}\left({{{{{\rm{AP}}}}}}\right)}(V)\equiv V\!/\!{V}_{{{{{{\rm{C}}}}}}0,{{{{{\rm{P}}}}}}({{{{{\rm{AP}}}}}})}$. *V*_C0,P(AP)_ denotes the intrinsic critical voltage for STT switching from parallel, P, to antiparallel, AP, states (AP to P states). Because the electric-field effect on anisotropy is governed by the applied voltage *V*, we use an expression based on voltage input rather than current input. Equation (2) contains so many unknown variables (*E*_0_, *H*_S_, *H*_*K*_^eff^(*V*), *V*_C0,P(AP)_, *n*_*H*_ and *n*_*I*_) that one cannot directly determine the exponents by only measuring RTN. Thus, in the following, we first separately determine *H*_*K*_^eff^(*V*) from a homodyne-detected FMR and the next *V*_C0_ from the STT switching probability. After that, we determine *H*_S_ and the exponents *n*_*H*_ and *n*_*I*_ from RTN measurement^34–38^ as a function of *V*.

## Electric-field effect on anisotropy field

**Fig. 2:**
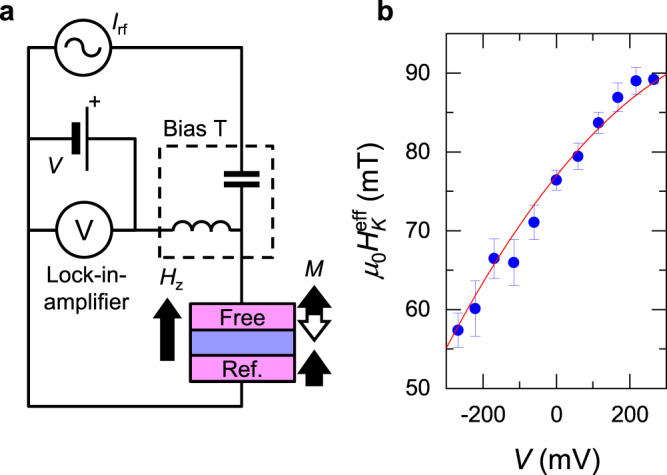
Homodyne-detected ferromagnetic resonance. **a** Electrical circuit for homodyne-detected ferromagnetic resonance (FMR). **b** DC bias voltage *V* dependence of effective anisotropy field (*μ*_0_*H*_*K*_^eff^) determined by FMR. Red curves are fitting with a quadratic function to the plots. The error bar shows the standard error of fitting Kittel’s resonant condition to the experimentally obtained resonant frequency versus perpendicular magnetic field *H*_*z*_.

Firstly, we determine *H*_*K*_^eff^(*V*) from homodyne-detected FMR under dc bias voltage^45,46^. Figure 2a shows the circuit configuration for the measurement. Homodyne-detected voltage spectra are measured while sweeping *H*_*z*_ at various frequencies and *H*_*K*_^eff^ is determined from the peak position (see Methods and Supplementary Information for details). We perform this measurement at various dc biases and obtain *H*_*K*_^eff^ *vs*. *V*, as shown in Fig. 2b. *H*_*K*_^eff^ changes nonlinearly with *V*. We fit a quadratic equation to the obtained dependence and determine the coefficients for constant, linear, and quadratic terms to be *μ*_0_*H*_*K*_^eff^(0) = 77.0 ± 0.5 mT, *μ*_0_d*H*_*K*_^eff^/d*V* = −57.8 ± 1.6 mT V^−1^, and *μ*_0_d^2^*H*_*K*_^eff^/d*V*^2^ = −49.9 ± 7.5 mT V^−2^ (*μ*_0_ is the permeability of vacuum). Note that the constant term represents the magnetic anisotropy field at zero bias whereas the linear and quadratic terms mainly originate from the electric-field modulation of anisotropy and an effect of Joule heating, respectively. The determined coefficients will be used in the analysis of RTN later.

## Intrinsic critical voltage for STT switching

**Fig. 3:**
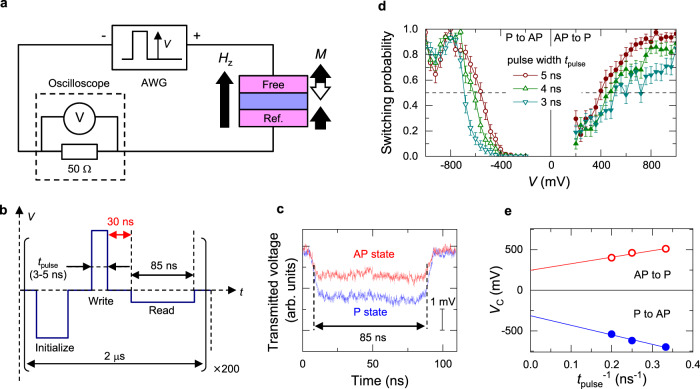
Spin-transfer torque switching. **a** Circuit configuration to measure spin-transfer-torque (STT) switching probability of low thermal stability factor MTJs. **b** Schematics of voltage waveform applied to MTJs. **c** Transmitted voltage monitored at oscilloscope during read sequence with a duration of 85 ns. **d** Switching probability as a function of pulse voltage amplitude *V* with different write pulse duration *t*_pulse_. **e** Switching voltage *V*_C_ as a function of *t*_pulse_^−1^. Lines are linear fits, whose intercept yields intrinsic critical voltages *V*_C0,P(AP)_.

Secondly, we determine *V*_C0_ for the STT switching. Because the fluctuation timescale of the studied system is on the order of milliseconds, we need to measure the junction state right after the switching pulse application when they are still non-volatile. To this end, we use a circuit configuration shown in Fig. 3a. This configuration is similar to that we used in our previous work to study the switching error rate^47^; a voltage waveform composed of initialisation, write, and read pulses [Fig. 3b] is applied to the MTJ by an arbitrary waveform generator, and the transmitted signal at the read pulse is monitored by a high-speed oscilloscope to identify the final state of magnetisation configuration. The typical transmitted signals for P and AP states are shown in Fig. 3c. A clear difference is observed in the amplitude of the transmitted signal for different configurations due to the tunnel magnetoresistance. Write and read pulses are separated by 30 ns, which is much shorter than the shortest relaxation time shown later (~0.3 ms), ensuring a sufficiently low read-error rate (unintentional switching probability before the read pulse) <10^−4^ (see Methods). The waveform is applied 200 times repeatedly and switching probability is evaluated.

Figure 3d shows the write pulse voltage dependence of the switching probability with different write pulse durations *t*_pulse_. The switching voltage *V*_C_, defined as the voltage at 50% probability, is plotted as a function of the inverse of *t*_pulse_ in Fig. 3e. In the precessional regime (typically *t*_pulse_ ≲ several nanoseconds) where the switching/non-switching is determined by an amount of transferred angular momenta, *V*_C_ is known to linearly depend on *t*_pulse_^−1^ and the intercept yields *V*_C0_^20,48^. From a linear fitting, *V*_C0,P_ and *V*_C0,AP_ are obtained as 313 ± 45 mV and −247 ± 42 mV, respectively.

## Random telegraph noise measurement to determine the switching exponents

**Fig. 4:**
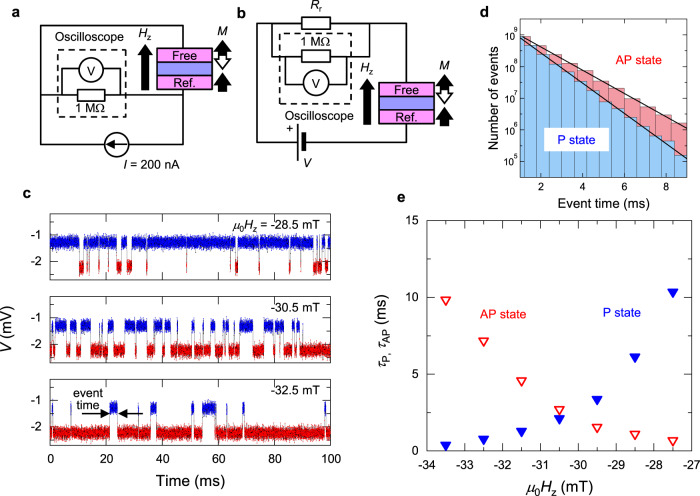
Random telegraph noise. **a**, **b** Circuit configuration to measure random telegraph noise (RTN) with *V* ~ 0 and *V* ≥ 25 mV, respectively. **c** Typical RTN signal monitored at the oscilloscope. **d** Histogram of event time (duration between two sequential switching events) for P and AP states for *μ*_0_*H*_*z*_ = −30.5 mT. **e** Expected switching time as a function of perpendicular magnetic field *H*_*z*_.

With the results above, we are now ready to determine the switching exponents, *n*_*H*_ and *n*_*I*_, from RTN measurement under various *V* and *H*_*z*_. Figures 4a, b show the circuit configurations for the measurement with *V* ~ 0 and |*V*| ≥ 25 mV, respectively. For *V* ~ 0, we apply a small direct current *I* = 200 nA and monitor *R* by an oscilloscope connected in parallel to the MTJ and probe the temporal magnetisation configuration. For |*V*| ≥ 25 mV, we monitor the divided voltage at reference resistor *R*_r_ serially connected to the MTJ using an oscilloscope. Note that *R*_r_ (= 470 Ω) is set to be much smaller than *R* to prevent a change in the electric-field effect on the magnetic anisotropy between P and AP states. Figure 4c shows typical results of RTN with various *H*_*z*_ and the definition of the magnetisation switching event time *t*. Figure 4d shows the distribution of the number of unique *t* for *μ*_0_*H*_*z*_ = −30.5 mT. As expected, the exponential distribution is confirmed, i.e., the number of events ∝ *τ*^−1^exp(-*t*/*τ*), indicating that the fluctuation is characterised by a Poisson process. From the fitting, expectation values of the event time for P and AP states, i.e., the relaxation time *τ*_P_ and *τ*_AP_, are obtained as a function of *H*_*z*_ as shown in Fig. 4e. Subsequently, *Δ*_P(AP)_ can be determined from the relation *τ* = *τ*_0_exp*Δ*_P(AP)_.

**Fig. 5:**
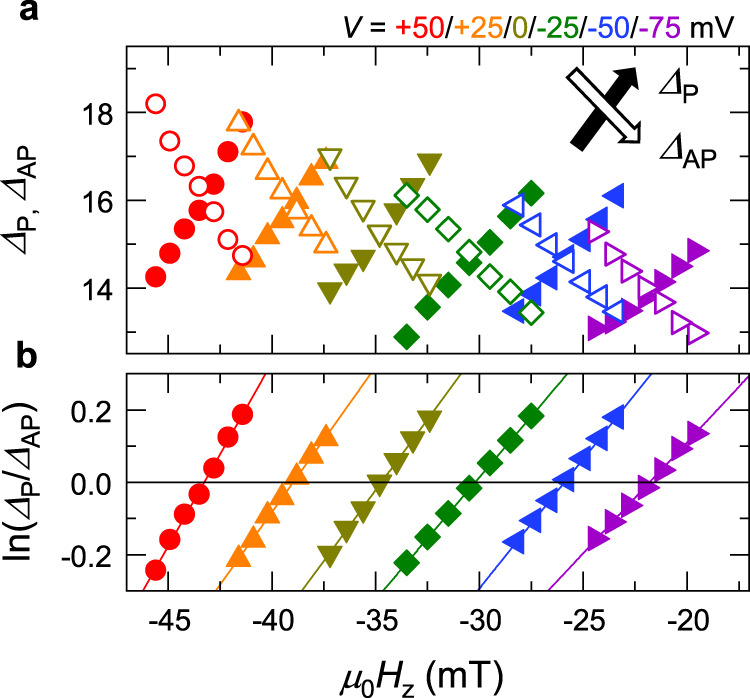
*Δ*_P_, *Δ*_AP_, and their ratio. **a** Thermal stability factors for P and AP states *Δ*_P_ and *Δ*_AP_ are determined by RTN measurement. **b** Natural log of their ratio as functions of perpendicular magnetic field *H*_*z*_ and *V*.

We measure *τ*_P(AP)_ and *Δ*_P(AP)_ for various *H*_*z*_ and *V*. Figure 5a shows the obtained *Δ*_P_ and *Δ*_AP_ as a function of *H*_*z*_ for various *V*. *Δ*_P(AP)_ increases (decreases) with increasing *H*_*z*_ for each *V*, as expected from the energy landscape modulation by *H*_*z*_. Also, the mean *Δ* gradually decreases with increasing *V*, which is also consistent with the trend of *H*_*K*_^eff^ shown in Fig. 2b. To derive *n*_*H*_ and *n*_*I*_, we then take the natural logarithm of the ratio between *Δ*_P_ and *Δ*_AP_, which can be expressed from Eq. (2) as

3 $${{{{{\rm{ln}}}}}}\frac{{\it\varDelta }_{{{{{{\rm{P}}}}}}}}{{\it\varDelta }_{{{{{{\rm{AP}}}}}}}}={n}_{H}{{{{{\rm{ln}}}}}}\frac{1+h}{1-h}+{n}_{I}{{{{{\rm{ln}}}}}}\frac{1-{v}_{{{{{{\rm{P}}}}}}}}{1-{v}_{{{{{{\rm{AP}}}}}}}}.$$

ln(*Δ*_P_/*Δ*_AP_) *vs*. *H*_*z*_ for each *V* is plotted in Fig. 5b. At a small perturbation limit, i.e., *h*, *v*_P_, *v*_AP_ ≪ 1, Eq. (3) is reduced to ln(*Δ*_P_/*Δ*_AP_) = 2*n*_*H*_*h* + *n*_*I*_(*v*_AP_ − *v*_P_); thus, the slope and intercept of the linear fit to the data shown in Fig. 5b give *n*_*H*_ and *n*_*I*_, respectively. One can see that the results are well fitted by the linear function, validating the employed model.

**Fig. 6:**
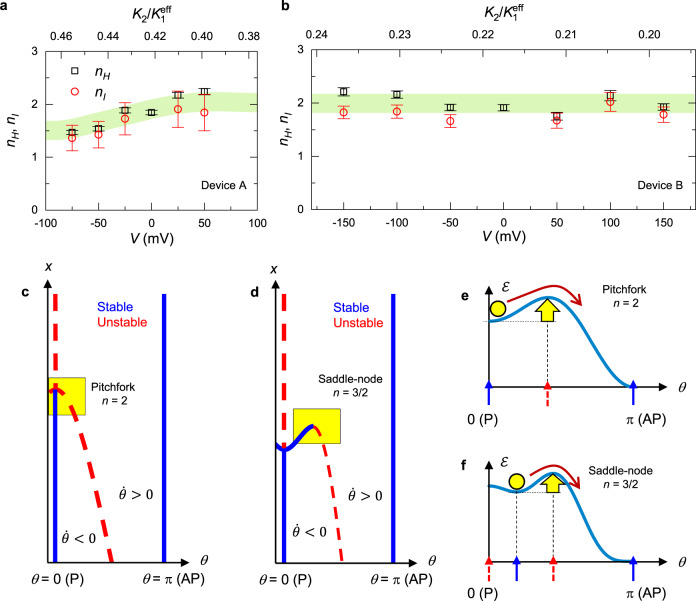
Experimentally determined switching exponents *n*_*H*_*n*_*I*_. **a**, **b** Field- and current-induced switching exponents *n*_*H*_ and *n*_*I*_ as a function of bias voltage *V* for **a** device A and **b** device B. Error bars show standard error propagated from ferromagnetic resonance measurement and critical current measurement. The green band is a guide to the eye. **c**, **d** Bifurcation diagrams of the d*θ*/d*t* projected upon (*θ*,*x*)(= (*θ*,*H*) or (*θ*,*I*)) for **c** *K*_2_/*K*_1_^eff^ < 0.25 and **d** *K*_2_/*K*_1_^eff^ > 0.25, where *K*_1_^eff^, *K*_2_, *θ*, *t*, *H*, and *I* are the first- and second-order effective anisotropy fields, polar angle of magnetisation vector, the time, perpendicular magnetic field, and current. **e**, **f** Schematics of energy barrier of the effective magnetic potential corresponding to the yellow region in **c** and **d**, respectively.

We analyse Fig. 5 with Eq. (3) and obtain *n*_*H*_ and *n*_*I*_ as a function of *V* for device A as shown in Fig. 6a. One can see that both *n*_*H*_ and *n*_*I*_ show similar values at each *V* and gradually decreases to about 1.5 with decreasing *V*. We perform the same procedure for the device B, whose properties are determined as *μ*_0_*H*_*K*_^eff^(0) = 129.0 ± 0.7 mT, *μ*_0_d*H*_*K*_^eff^/d*V* = −61.7 ± 2.3 mT V^−1^, *μ*_0_d^2^*H*_*K*_^eff^/d*V*^2^ = −58 ± 13 mT V^−2^, *V*_C0,P_ = 672 ± 4 mV and *V*_C0,AP_ = −541 ± 2 mV. The obtained *n*_*H*_ and *n*_*I*_ are shown in Fig. 6b. At *V* = 0, the two devices show the same value for *n*_*H*_ within experimental inaccuracy. Also, both *n*_*H*_ and *n*_*I*_ of device B show similar values with each other as in device A. However, in contrast to device A, they do not show meaningful variations at around 2 with *V*.

## Discussion

As shown above, we have found that *n*_*H*_ and *n*_*I*_ show virtually the same value with each other for both devices A and B. Also, they are almost constant at around 2.0 for device B whereas change from 2.0 to 1.5 with *V* for device A. The main difference between devices A and B is *t*_CoFeB_, which manifests in a difference in *μ*_0_*H*_*K*_^eff^(0) [77.0 ± 0.5 mT for device A and 129.0 ± 0.7 mT for device B]. In the following, we will discuss the mechanism that can account for the obtained results in the context of the energy landscape and its bifurcation.

In systems with uniaxial anisotropy where the magnetic field is applied along the easy axis where the macrospin approximation holds, Brown derived *n*_*H*_ = 2 in a high barrier region, *E*_0_ ≳ *k*_B_*T*, using Kramers’ analysis on the Fokker–Planck equation that is equivalent to the Landau–Lifshitz–Gilbert (LLG) equation with the Langevin term^3^. Taking into consideration the second-order anisotropy, where the magnetic anisotropy energy density is given by ${{{{{\mathcal{E}}}}}}$ = *K*_1_^eff^sin^2^*θ* + *K*_2_sin^4^*θ*, *n*_*H*_ was pointed out to vary with *K*_2_/*K*_1_^eff^ ^7^, where *K*_1_^eff^, *K*_2_ and *θ* are the first- and second-order effective anisotropy fields and polar angle of magnetisation vector, respectively. In the CoFeB/MgO system, positive voltage, which decreases electron density at the interface, was found to increase *K*_1_^eff^, while keeping *μ*_0_*H*_*K*2_ (≡*μ*_0_*K*_2_/4*M*_S_) constant at around 45 mT^46,49,50^. Accordingly, as shown in the upper axes of Fig. 6a, b, in the present cases, *K*_2_/*K*_1_^eff^ is calculated to be around 0.22 for device B whereas it increases up to 0.45 for device A. The numerical calculation, assuming material parameters of magnetic recording media (*Δ*_0_ ≳ 60), shows that *n*_*H*_ decreases from 2.0 to 1.5 in the range of *K*_2_/*K*_1_^eff^ from 0 to 0.25^7^. In general, a dynamical system with pitchfork bifurcation leads to the switching exponent of *n*_*x*_ = 2, while saddle-node bifurcation results in *n*_*x*_ = 3/2. Note that the aforementioned magnetic energy density ${{{{{\mathcal{E}}}}}}$ gives the LLG equation d*θ*/d*t* = *f*(*θ*,*H*_*z*_) = −*αγμ*_0_[(2*K*_1_^eff^/*M*_S_)cos*θ* − (4*K*_2_/*M*_S_)cos^3^*θ* − *H*_*z*_]sin*θ*. We show *f*(*θ*,*H*_*z*_) takes two types of local bifurcations: pitchfork bifurcation appears at *K*_2_/*K*_1_^eff^ < 0.25, while saddle-node bifurcation at *K*_2_/*K*_1_^eff^ > 0.25 as shown in Fig. 6c, d, respectively [see Supplementary Information in detail]. Thus, the experimentally observed transition of the switching exponents is attributed to the transition of the bifurcation of the potential landscape through the modulation of *K*_2_/*K*_1_^eff^. However, the experiment shows the transition of *n*_*H*_ at *K*_2_/*K*_1_^eff^ ≈ 0.45, which is larger than that expected by the macrospin model (*K*_2_/*K*_1_^eff^ = 0.25). This deviation implies that the local bifurcation of the magnetic potential and the resultant *n*_*H*_ in the real MTJ device is more insensitive to higher-order anisotropy field *K*_2_ than the macrospin limit, for example, due to the micromagnetic effects.

Regarding *n*_*I*_, some theoretical studies derived 1 by considering a fictitious temperature in LLG equation with the Langevin term^20,21^, whereas others derived 2 from an analysis of the Fokker–Planck equation^22–24^. Matsumoto et al. pointed out that *n*_*I*_ rapidly decreases from 2 to 1.4 with increasing *K*_2_/*K*_1_^eff^ from 0 to ~0.25^51^. Experimentally, some assumed 1^25,27–30,36,39^ whereas others assumed 2^26,31–33^, and importantly no studies access the number. The present experimental results support the scenario of Matsumoto et al., but, similarly to *n*_*H*_, the reduction of *n*_*I*_ is more moderate than the theoretical prediction. This fact indicates that the mechanism for *n*_*H*_ could be also applicable for the case with STT perturbation as well. Another important implication of our results is that, despite the non-conservative nature of STT, the pseudo energy landscape under STT can be investigated through the switching exponents. LLG equation with STT *τ*_STT_ can be represented as d*θ*/d*t* = *f*(*θ*,*x*) = {−*αγμ*_0_[(2*K*_1_^eff^/*M*_S_)cos*θ* − (4*K*_2_/*M*_S_)cos^3^*θ*] + *τ*_STT_}sin*θ*. Since *n*_*H*_ and *n*_*I*_ show virtually the same value for all *K*_2_/*K*_1_^eff^ conditions, meaning that *f*(*θ*,*τ*_STT_) takes the same local bifurcation type as that for *f*(*θ*,*H*), our experiment reveals that in MTJ devices with perpendicular easy axis, the magnetic field and the STT effectively similarly modulate the energy landscape.

In summary, this work has experimentally revealed the hitherto-inaccessible representation of thermally-activated switching rate under field and STT, using a relevant material system for applications. The obtained results could allow for sophisticated engineering of non-volatile memory and unconventional computing hardware. Through the switching exponents, we have also accessed the local bifurcation of energy landscape under STT, and have found that, despite the qualitative difference between magnetic field and STT, their effect on the energy landscape is equivalent in the case of perpendicular MTJ. This work has also demonstrated that superparamagnetic tunnel junctions and analysis of their local bifurcation can serve as a versatile tool to investigate unexplored physics relating to thermally-activated phenomena in general with various configurations and external perturbations.

## Methods

### Sample preparation

Stacks with Ta(5)/Pt(5)/[Co(0.3)/Pt(0.4)]_7_/Co(0.3)/Ru(0.45)/[Co(0.3)/Pt(0.4)]_2_/Co(0.3)/Ta(0.3)/CoFeB(1.0)/MgO(1.0)/CoFeB(*t*_CoFeB_)/Ta(5)/Ru(5) (numbers in parenthesis are thickness in nm) were deposited by dc/rf magnetron sputtering on a sapphire substrate. The nominal CoFeB thicknesses *t*_CoFeB_ = 1.88 nm (device A) and 1.82 nm (device B). After the deposition, the stacks were processed into MTJs by a hard-mask process with electron-beam lithography, followed by annealing at 300 °C under a perpendicular magnetic field of 0.4 T for 1 h. The resistance (*R*)-area (*A*) product (*RA*) was determined from the physical size determined from scanning electron microscopy observation and measured resistance for large devices with diameter *D* > 45 nm. The resistance-area product of device A (device B) is 5.5 Ωμm^2^ (8.1 Ωμm^2^), and the tunnel magnetoresistance ratio is 73% (74%). The nominal thickness of the MgO is 1.0 nm for both devices, and the difference of the *RA* corresponds to the ~7% variation of the actual thickness due to the process variations between the two runs. *D* of devices A and B are determined from their resistance and *RA* to be *D* = 34 and 28 nm, respectively.

### Homodyne-detected ferromagnetic resonance (FMR)

With the circuit shown in Fig. 2a, homodyne-detected voltage-*H*_*z*_ spectra were measured at various frequencies. As shown in previous papers, the spectra were well fitted by the Lorentz function and peak position was determined by the fitting^45,46^. From resonance frequency *f*_r_ vs. *H*_*z*_, the effective anisotropy field *H*_*K*_^eff^ was determined while assuming a constant second-order anisotropy field *μ*_0_*H*_*K2*_ = 45 mT^46,49,50^ (*μ*_0_ is the permeability of vacuum). The measurement was performed at various dc biases at AP configuration and obtained *H*_*K*_^eff^ vs. *V*, as shown in Fig. 2b. A quadratic equation was fitted to the obtained dependence and the coefficients for constant, linear and quadratic terms were determined. Note that, in the error of *H*_*K*_^eff^, we have included the effect of *H*_*K*_^eff^ difference in P and AP configurations due to the different device resistances and resultant Joule heatings in these configurations under the identical bias voltage.

### Switching probability measurement

With the circuit shown in Fig. 3a, the switching probability was measured as functions of write pulse voltage amplitude and duration to determine intrinsic critical voltage *V*_C0_. A voltage waveform composed of initialisation (0.45 V/300 ns), write (amplitude *V*_write_/duration *t*_pulse_), and read (*V*_read_ = 0.15 V/75 ns) pulses as shown in Fig. 3b, was generated by an arbitrary waveform generator (AWG). Both the interval of initialisation/write and write/read pulses were 30 ns, which is much shorter than the shortest relaxation time measured here (~0.3 ms), ensuring a sufficiently low read-error rate due to unintentional switching probability before the read pulse, exp(−30 ns/0.3 ms) $\le$ 10^−4^. Single-shot-transmitted voltage for write pulse was monitored to determine the magnetisation configuration; the transmitted voltage is ~2*Z*_0_*V*_read_/(*R* + *Z*_0_), where *Z*_0_ is characteristic impedance 50 Ω, and due to the tunnel magnetoresistance, the transmitted voltage changes with magnetisation configuration. The typical transmitted signal for P and AP states is shown in Fig. 3c. Transmitted signals for 5 ns (between 15 and 20 ns in Fig. 3c) were averaged. Its averaged value 〈*V*〉 and standard error 〈(*V*-〈*V*〉)^2^〉^0.5^/*N*^ 0.5^ for P and AP states were 2.44 ± 0.03 mV and 1.40 ± 0.03 mV, respectively (*N* is averaged points; 20 Gbit/s × 5 ns duration = 100 points), ensuring low read-error rate due to misassignment of the magnetisation configuration^47^. As shown in Fig. 3d, switching probability as a function of the voltage amplitude *V* at MTJ with write pulse duration *t*_pulse_ from 1 to 5 ns was measured. The probability of the switching was determined from 200 times measurement. The switching measurement was conducted under *H*_*z*_ of the stray field *H*_S_ which was determined from the random telegraph noise measurement. Note that the anomaly of the switching probability at *V* ~ −900 mV can be attributed to a change of the magnetic easy axis through the electric-field effect on the magnetic anisotropy, which is reported in previous works^52^. In addition, the slope of the switching probability at *P*_sw_ ~ 0.5 for P to AP switching increases with decreasing the pulse duration, which is opposite to the thermally-stable MTJs. The decreases of the effective field and the thermal stability factor through the electric-field effect on magnetic anisotropy reasonably explain the behaviour.

### Random telegraph noise (RTN)

With the circuit shown in Fig. 4a, the RTN signal of the MTJs for *V* ~ 0 was measured. Small direct current *I* = 200 nA was applied and *R* was monitored by an oscilloscope connected in parallel to the MTJ to probe the temporal magnetisation configuration. The voltage applied to MTJ here was up to 2.5 mV (5 mV) for device A (device B), which is small enough to prevent major voltage/current-induced effects in MTJs focused here. If one utilises the same circuit, the applied voltage for P and AP states varies by a factor of about 1.7 due to the tunnel magnetoresistance effect. Therefore, to prevent variation of effective anisotropy field for P and AP states, the circuit shown in Fig. 4b was utilised for |*V*| ≥ 25 mV. Direct voltage *V* to the MTJ was applied and divided voltage at reference resistor *R*_r_ connected in serial to the MTJ was monitored using the oscilloscope. For measuring RTN on device A (device B), with setting *R*_r_ = 0.47 kΩ (1 kΩ) much smaller than *R*, variation of applied voltages between P and AP states was prevented.

### Attempt frequency

In determining *Δ* with the random telegraph noise measurement, Néel–Arrhenius raw *τ*_P(AP)_ = *τ*_0_exp*Δ*_P(AP)_ with attempt frequency *τ*_0_ of 1 ns was assumed. This assumption is widely adopted because *τ* is an exponential function of *Δ* and the value of *τ*_0_ does not affect the estimated *Δ*, and *τ*_0_ ranges between 0.1 and 10 ns. According to Brown’s calculation with Kramer’s method on the Fokker–Planck equation^3^, the attempt frequency *τ*_0_ of the magnetic materials with uniaxial perpendicular magnetic anisotropy is [2*αγμ*_0_*H*_*K*_^eff^(1 − *h*^2^)(1 + *h*)]^−1^(π/*Δ*_0_)^0.5^ under large barrier approximation *Δ*_0_$\gg$1, where *α*, *γ*, and *μ*_0_ are damping constant 0.006, gyromagnetic ratio, and permeability of vacuum, respectively. In our devices, the Brown’s attempt frequency above is derived to be 1.1 and 2.4 ns for device A and device B, respectively. Thus, the switching time *τ* vs. thermal stability factor *Δ* of device A should be well described by the Néel–Arrhenius law with *τ*_0_ = 1 ns.

## Supplementary information

Supplementary Information

## Data Availability

The data that support the plots within this paper have been deposited in Zenodo at https://zenodo.org/record/6767828^53^.
