# Supplementary material for: Local bifurcation with spin-transfer torque in superparamagnetic tunnel junctions
Source: Nat Commun. 2022 Jul 14;13:4079. doi: 10.1038/s41467-022-31788-1 (PMC9283488; doi:10.1038/s41467-022-31788-1)
Supplement: Supplementary file 1 — Supplementary Information [file 41467_2022_31788_MOESM1_ESM.pdf]

Local bifurcation with spin-transfer torque in superparamagnetic tunnel junctions

Takuya Funatsu[1], Shun Kanai[1,2,3,4*], Jun'ichi Ieda[1,5], Shunsuke Fukami[1,4,6,7,8], and Hideo Ohno[1,4,6,7]

[1] *Laboratory for Nanoelectronics and Spintronics, Research Institute of Electrical Communication, Tohoku University, Sendai, Japan*
[2] *PRESTO, Japan Science and Technology Agency, Kawaguchi, Japan*
[3] *Division for the Establishment of Frontier Sciences, Tohoku University, Sendai, Japan*
[4] *Center for Science and Innovation in Spintronics, Tohoku University, Sendai, Japan*
[5] *Advanced Science Research Center, Japan Atomic Energy Agency, Tokai, Japan*
[6] *WPI-Advanced Institute for Materials Research, Tohoku University, Sendai, Japan*
[7] *Center for Innovative Integrated Electronic Systems, Tohoku University, Sendai, Japan*
[8] *Inamori Research Institute for Science, Kyoto, Japan*

**e-mail: skanai@tohoku.ac.jp*

Supplemental information

S1. Determination of effective anisotropy field with bias dependent ferromagnetic resonance (FMR)

To determine the anisotropy field with bias voltage V, we measure the bias-dependent FMR in the MTJ by utilizing the circuit shown in Fig. 2a. Rf power with -15 dBm is applied to MTJ through the rf port of the bias-tee, and the rectified signal is measured through its dc port. We modulate the rf power with 273 Hz, and the homodyne detected signal V_{dc} is collected at the lock-in amplifier[1]. Figure S1a shows the homodyne-detected voltage V_{dc} spectra dependence on perpendicular magnetic field H_z with zero bias voltage V under various rf frequencies f for device B. The spectrum shows dominant contribution of the anti-symmetric Lorentz function, indicating FMR is mainly induced by an electric-field effect on magnetic anisotropy and/or field-like torque[2,3]. We fit the anti-symmetric Lorentz function to experimental data and obtain the resonance field H_{r}. The open circle plot in Fig. S1b shows $(H_{\mathrm{r}} - H_{\mathrm{S}})$ *vs.* f at $V = 0$ (H_{S} is the stray field from the reference layer determined by the random-

telegraph-noise measurement), showing a clear linear relationship between them. The magnetic energy density $\mathcal{E}$ with Zeeman energy and uniaxial magnetic anisotropy energy is[3,4]

$$\begin{aligned}\mathcal{E} &= -M_{\mathrm{S}}(H_z - H_{\mathrm{S}})\cos\theta + K_1^{\mathrm{eff}}\sin^2\theta + K_2\sin^4\theta \\ &= -M_{\mathrm{S}}(H_z - H_{\mathrm{S}})\cos\theta + \left(K_1 - \frac{M_{\mathrm{S}}^2}{2\mu_0}\right)\sin^2\theta + K_2\sin^4\theta \\ &= -M_{\mathrm{S}}(H_z - H_{\mathrm{S}})\cos\theta - \left(-\frac{M_{\mathrm{S}}^2}{2\mu_0} + K_1 + 2K_2\right)\cos^2\theta + K_2\cos^4\theta + K_1 + K_2 \\ &= -M_{\mathrm{S}}(H_z - H_{\mathrm{S}})\cos\theta - \frac{M_{\mathrm{S}}H_{K1}^{\mathrm{eff}}}{2}\cos^2\theta + \frac{M_{\mathrm{S}}H_{K2}}{4}\cos^4\theta + K_1 + K_2,\end{aligned} \tag{S1}$$

where μ_0, θ, M_{S}, K_1, K_2, H_{K1}^{eff}, and H_{K2} are permeability of vacuum, the angle between magnetization and film normal, spontaneous magnetization, first-order magnetic anisotropy constant, second-order magnetic anisotropy constant, first-order effective anisotropy field, and second-order anisotropy field, respectively. The resonance frequency around $\theta = 0$ is

$$f = \frac{\gamma\mu_0}{2\pi}(H_{\mathrm{r}} - H_{\mathrm{S}} + H_{K1}^{\mathrm{eff}} - H_{K2}), \tag{S2}$$

where γ is the gyromagnetic ratio. From equation (S2) and intercept of the $(H_{\mathrm{r}} - H_{\mathrm{S}})$ *vs.* f, $\mu_0(H_{K1}^{\mathrm{eff}} - H_{K2})$ is determined to be 108.5±1.6 mT. Effective anisotropy field H_K^{eff} defined as $\mathcal{E}(\frac{\pi}{2}) - \mathcal{E}(0) = \frac{1}{2}M_{\mathrm{S}}H_K^{\mathrm{eff}}$ is $H_K^{\mathrm{eff}} = H_{K1}^{\mathrm{eff}} - \frac{H_{K2}}{2}$. According to previous reports[3-5], in CoFeB/MgO systems, $\mu_0 H_{K2}$ is 45 mT and does not vary with electric field nor CoFeB thickness. Thus, assuming this H_{K2} value, H_K^{eff} is determined from $H_{K1}^{\mathrm{eff}} - H_{K2}$ obtained from FMR; $\mu_0 H_K^{\mathrm{eff}}$ at $V = 0$ is determined to be 131.0±1.6 mT. Figure S1c shows the bias dependence of the FMR spectra at a frequency of 4 GHz. The bias voltage induces a clear variation of the spectra from the anti-symmetric Lorentz function to the symmetric Lorentz function, which is explained by the bias-dependent reflection coefficient spectra[3]. With the same procedure as $V = 0$, the resonance field *vs.* rf frequency is measured for various V [Fig. S1b], and H_K^{eff} as a function of V is shown in Fig. S1d. As shown in the main body, H_K^{eff} changes non-linearly with V. We fit a quadratic equation to the obtained dependence and

determine the coefficients for constant, linear, and quadratic terms as $\mu_0 H_K^{\mathrm{eff}}(0) = 129.0\pm0.7$ mT, $\mu_0 \frac{\mathrm{d}H_K^{\mathrm{eff}}}{\mathrm{d}V} = -61.7\pm2.3$ mT/V, $\mu_0 \frac{\mathrm{d}^2 H_K^{\mathrm{eff}}}{\mathrm{d}V^2} = -58\pm13$ mT/V^2.

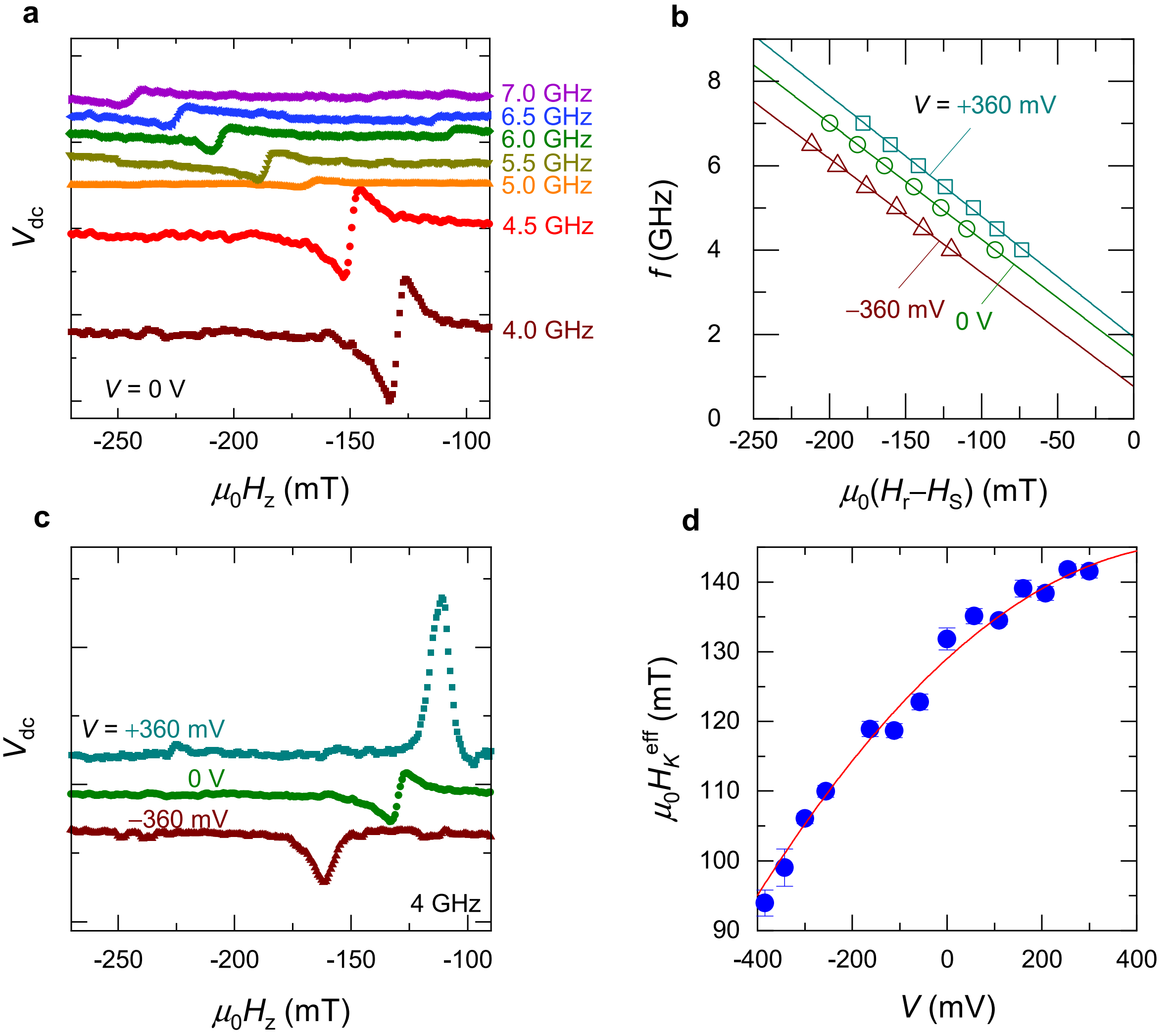

Fig. S1 | Homodyne detected ferromagnetic resonance. a, homodyne-detected voltage V_{dc} spectra on a perpendicular magnetic field H_z with zero bias voltage V under various rf frequencies f. **b**, Peak frequency f as a function of resonance frequency H_{r} subtracted by stray field from the reference layer H_{S} with various V. **c**, V_{dc} spectra on H_z at 4 GHz with various V. **d**, Effective anisotropy field H_K^{eff} as a function of V.

S2. Types of local bifurcations and their switching exponents

In this section, we summarise the typical local bifurcations and investigate their corresponding energy landscape and switching exponents for the external input. First, we start from a general dynamical equation, which is expressed as

$$\frac{\mathrm{d}\Theta}{\mathrm{d}t} = f(\Theta, x), \tag{S3}$$

where Θ is a state variable (magnetisation direction, position of the particle, unreacted chemical amount, *etc*...), x is external input [6]. We assume the Θ changes according to the potential gradient, namely, $f(\Theta, x) = -\frac{\partial\phi}{\partial\Theta}$. Here, we investigate energy barrier E of the potential landscape ϕ with several types of the local bifurcations relating to magnetic systems used in the main text.

(I) Subcritical pitchfork bifurcation

A dynamical equation

$$\frac{\mathrm{d}\Theta}{\mathrm{d}t} = f(\Theta, x) = \Theta^3 + (x - x_0)\Theta \tag{S4}$$

gives a bifurcation shown in Fig. S2a, where the x_0 is the threshold input. The condition $\frac{\mathrm{d}\Theta}{\mathrm{d}t} = 0$ is shown by the lines: stable and unstable conditions are shown by the blue solid lines and the red dashed lines, respectively. This type of bifurcation is called subcritical pitchfork bifurcation. From $f(\Theta, x) = -\frac{\partial U}{\partial\Theta}$, potential landscape $\phi = -\frac{1}{4}\Theta^4 - \frac{1}{2}(x - x_0)\Theta^2$. At $x > x_0$, ϕ is convex upwards with Θ and has one unstable point with $\frac{\mathrm{d}\Theta}{\mathrm{d}t} = 0$ at $\Theta = 0$ as shown in Fig. S2b. At $x < x_0$, on the other hand, the potential landscape has stable point at $\Theta = 0$, and two unstable points with $\frac{\mathrm{d}\Theta}{\mathrm{d}t} = 0$ at $x = x_0 - \Theta^2$ as shown in Fig. S2c. Energy barrier $E(x)$ is calculated to be $E(x) = \phi(\Theta = \sqrt{x_0 - x}) - \phi(\Theta = 0) = \frac{1}{4}(x_0 - x)^2$, and proportional to $\left(1 - \frac{x}{x_0}\right)^2$, indicating the switching exponent to the external field x is $n_x = 2$.

(II) Supercritical pitchfork bifurcation

Another example of the typical dynamical equation

$$\frac{\mathrm{d}\Theta}{\mathrm{d}t} = f(\Theta, x) = -\Theta^3 + (x - x_0)\Theta \tag{S5}$$

gives a local bifurcation structure shown in Fig. S2d, which is called supercritical pitchfork bifurcation. The structure is similar to the subcritical pitchfork bifurcation, but stable and unstable positions are exchanged with each other. The potential can be expressed as $\phi = \frac{1}{4}\Theta^4 - \frac{1}{2}(x - x_0)\Theta^2$. At $x > x_0$, ϕ has an unstable point at $\Theta = 0$, and two stable points at $x = x_0 - \Theta^2$ as shown in Fig. S2e. At $x < x_0$, ϕ is convex upwards and has one unstable point at $\Theta = 0$ as shown in Fig. S2f. The energy barrier at $x > x_0$ is calculated in the same manner as the case for the subcritical pitchfork bifurcation; $E \propto \left(1 - \frac{x}{x_0}\right)^2$ offers the switching exponent $n_x = 2$.

(III) Saddle-node bifurcation

The last example of the dynamical equation here is

$$\frac{\mathrm{d}\Theta}{\mathrm{d}t} = f(\Theta, x) = \Theta^2 + (x - x_0), \tag{S6}$$

which gives a bifurcation shown in Fig. S2g, which is called saddle-node bifurcation. This offers the potential landscape of $\phi = -\frac{1}{3}\Theta^3 - (x - x_0)\Theta$. At $x > x_0$, the potential landscape monotonically decreases with the increase of Θ (Fig. S2h). At $x < x_0$, the potential landscape has a stable point at $\Theta = -\sqrt{x_0 - x}$, and an unstable point at $\Theta = \sqrt{x_0 - x}$. The barrier height is $E(x) = \phi(\Theta = \sqrt{x_0 - x}) - \phi(\Theta = -\sqrt{x_0 - x}) = \frac{4}{3}(x_0 - x)^{\frac{3}{2}}$, and $E \propto \left(1 - \frac{x}{x_0}\right)^{\frac{3}{2}}$. Thus, the switching exponent for saddle-node bifurcation is $n_x = \frac{3}{2}$.

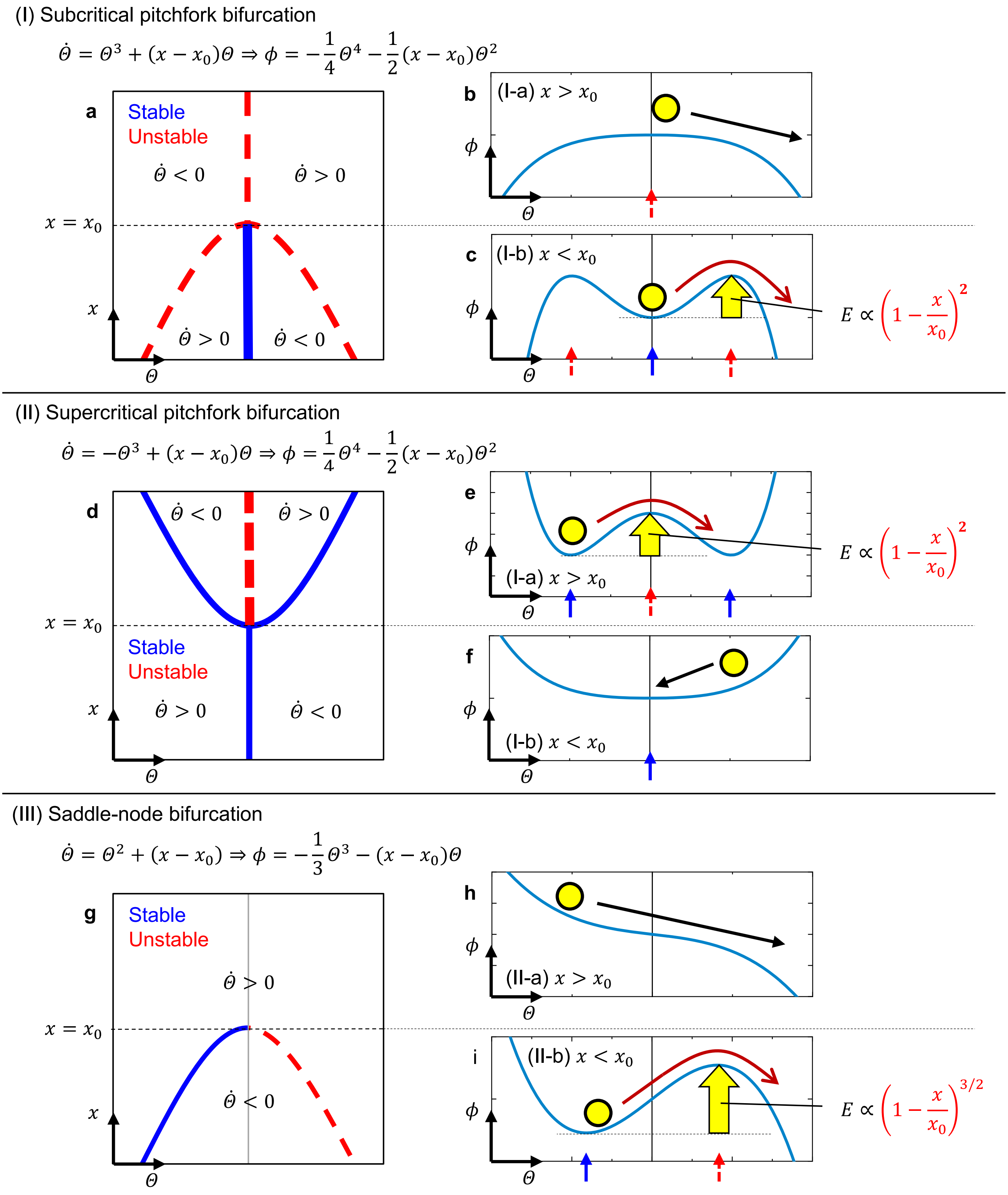

Fig. S2 | Types of local bifurcations and their switching exponents. a, An example of subcritical pitchfork bifurcation; the sign of time derivative of state variable $\dot{\Theta}$ as functions of external

input x and Θ for $\dot{\Theta} = \Theta^3 + (x - x_0)\Theta$, where x_0 is the threshold input. Stable and unstable points are shown by the lines with blue solid lines and red dashed lines, respectively. The corresponding potential ϕ as a function of Θ **b,** at $x > x_0$ and **c**, at $x < x_0$. **d**, Supercritical pitchfork bifurcation; $\dot{\Theta}$ as functions of x and Θ for $\dot{\Theta} = -\Theta^3 + (x - x_0)\Theta$. The corresponding ϕ as a function of Θ **e,** at $x > x_0$ and **f**, at $x < x_0$. **g**, Saddle-node bifurcatoin; $\dot{\Theta}$ as functions of x and Θ for $\dot{\Theta} = \Theta^2 + (x - x_0)$. The corresponding ϕ as a function of Θ **h,** at $x > x_0$ and **i**, at $x < x_0$.

S3. Local bifurcation of magnetization dynamics with uniaxial magnetic anisotropy under the macrospin limit

The Landau-Lifshitz-Gilbert (LLG) equation under thermal agitation is

$$\frac{\mathrm{d}\vec{m}}{\mathrm{d}t} = -\gamma\vec{m}\times\mu_0\left(\frac{1}{M_\mathrm{S}}\frac{\partial\mathcal{E}}{\partial\vec{m}}+\vec{h}_\mathrm{T}\right)+\tau_\mathrm{STT}\vec{m}\times(\vec{m}\times\vec{m}_\mathrm{R})+\alpha\vec{m}\times\frac{\mathrm{d}\vec{m}}{\mathrm{d}t}, \tag{S7}$$

where $\vec{m}$ is a normalised magnetisation vector defined by polar and azimuthal angles (θ,φ) as $(\sin\theta\cos\varphi\,,\sin\theta\sin\varphi\,,\cos\theta)$, *t* the time, γ the gyromagnetic ratio, μ_0 the permeability of vacuum, $\mathcal{E}(\theta,\varphi)$ the magnetic energy density, $\vec{h}_\mathrm{T}$ the thermal field vector defined by $\langle\vec{h}_\mathrm{T}\rangle=\vec{0}$ and $\langle h_{\mathrm{T},i}h_{\mathrm{T},j}\rangle=\frac{2\alpha k_\mathrm{B}T}{\gamma\mu_0 M_\mathrm{S}V\Delta t}\delta(i,j)$, with $k_\mathrm{B}T$ being the thermal energy, $M_\mathrm{S}V$ the magnetic moment of the free layer, $\delta(i,j)$ the Kronecker's delta with $\{i,j\}=\{\theta,\varphi\}$, Δt the time step, τ_STT the spin-transfer torque proportional to V, $\vec{m}_\mathrm{R}$ the normalised magnetisation vector of the reference layer, and α the damping constant. For the magnetic potential with uniaxial symmetry with $\mathcal{E}(\theta)$ and $\vec{m}_\mathrm{R}=\hat{\vec{z}}$, the LLG equation without thermal agitation is reduced to

$$\frac{\mathrm{d}\theta}{\mathrm{d}t}=\left(\frac{\alpha\gamma\mu_0}{M_\mathrm{S}}\frac{\partial\mathcal{E}}{\partial m_z}+\tau_\mathrm{STT}\right)\sin\theta\equiv f(\theta,H_z,\tau_\mathrm{STT}) \tag{S8}$$

because of the symmetry. This section aims to investigate the local bifurcations of the Eq. (S8).

We assume $\mathcal{E}$ is composed of first and second-order magnetic anisotropy and external magnetic field along *z*-direction as $\mathcal{E}(\theta)=K_1^\mathrm{eff}\sin^2\theta+K_2\sin^4\theta-M_\mathrm{S}H_z\cos\theta$ (Eq. (S1) with $H_\mathrm{S}=0$). Then,

$$\begin{aligned}\frac{\mathrm{d}\theta}{\mathrm{d}t}&=f(\theta,x)\\&=[-\alpha\gamma\mu_0(H_{K1}^\mathrm{eff}\cos\theta-H_{K2}\cos^3\theta+H_z)+\tau_\mathrm{STT}]\mathrm{sin}\theta\\&\equiv(-k_1\cos\theta+k_2\cos^3\theta+x)\mathrm{sin}\theta.\end{aligned} \tag{S9}$$

x is the external input from the field and current defined as $x=-\alpha\gamma\mu_0H_z+\tau_\mathrm{STT}$. $f(\theta,x)$ is not as simple as those shown in the previous section and its resultant global bifurcation is more complicated

than those shown in Fig. S2. However, we show that at around a critical point relating to the magnetisation switching, it can be classified by three local bifurcations shown in the previous section.

At $\theta \approx 0$, $f(\theta, x)$ is expanded as

$$\frac{\mathrm{d}\theta}{\mathrm{d}t} = f(\theta, x) \approx \frac{1}{2}(k_1 - 3k_2)\theta^3 + (x - x_0)\theta, \tag{S10}$$

indicating that the sign of the $k_1 - 3k_2$ determines the types of the bifurcations between the subcritical pitchfork bifurcation and the supercritical pitchfork bifurcation with $x_0 = k_1 - k_2 = \alpha\gamma\mu_0(H_{K1}^{\mathrm{eff}} - H_{K2})$ being the threshold input.

(i) For $k_1 > 3k_2 \left(\Longleftrightarrow H_{K1}^{\mathrm{eff}} > 3H_{K2} \Longleftrightarrow \frac{K_2}{K_1^{\mathrm{eff}}} < \frac{1}{4}\right)$: subcritical pitchfork bifurcation $(\theta, x) = (0, x_0)$

The local bifurcation of the $f(\theta, x)$ is shown in Fig. S3a. At around $(\theta, x) = (0, x_0)$ (point A in Fig. S3a: around P state and threshold input), it shows the subcritical pitchfork bifurcation. As shown in Fig. S3c, at $x > x_0$, there are no potential barriers and magnetisation stabilises to $\theta = \pi$ (AP state). At $x > x_0$, the switching occurs within this bifurcation and stabilises to $\theta = \pi$ (AP state). There is an unstable point between P and AP states at $\theta = \theta_0$, and the barrier height of magnetic potential is, $E = \mathcal{E}(\theta = \theta_0, x) - \mathcal{E}(\theta = 0, x)$, which is proportional to $\left(1 - \frac{x}{x_0}\right)^2$ and whose exponent is $n_x = 2$ from the discussion in the previous section.

(ii) For $k_1 < 3k_2 \left(\Longleftrightarrow H_{K1}^{\mathrm{eff}} < 3H_{K2} \Longleftrightarrow \frac{K_2}{K_1^{\mathrm{eff}}} > \frac{1}{4}\right)$: supercritical pitchfork bifurcation at $(\theta, x) = (0, x_0)$, saddle-node bifurcation at $(\theta, x) = (\theta_1, x_1)$

The local bifurcation of the $f(\theta, x)$ is shown in Fig. S3b. From Eq. (S10), the supercritical pitchfork bifurcation is obtained at around $(\theta, x) = (0, x_0)$ shown as A' point in Fig. S3b. In addition, Eq. (S9) gives another characteristic structure relating to the switching; at around B point ($(\theta, x) =$

$\left(\theta_1 \equiv \mathrm{acos}\sqrt{\frac{k_1}{3k_2}}, x_1 \equiv \frac{2\sqrt{3}}{9}k_1^{\frac{3}{2}}k_2^{-\frac{1}{2}}\right)$), a saddle-node bifurcation emerges as shown in the figure. Eq. (S9) is expanded at around $\theta = \theta_1$ as

$$\frac{\mathrm{d}\theta}{\mathrm{d}t} = f(\theta, x) \approx \frac{1}{2}(3k_2 - 1)\sin 2\theta_1 (\theta - \theta_1)^2 + (x - x_1)\sin\theta_1, \qquad \text{(S11)}$$

and thus, the local bifurcation coincides with the Eq. (S6). Especially at $x_0(= k_1 - k_2) < x < x_1 \left(\equiv \frac{2\sqrt{3}}{9}k_1^{\frac{3}{2}}k_2^{-\frac{1}{2}}\right)$, the switching takes place within saddle-node bifurcation, $E \propto \left(1 - \frac{x}{x_1}\right)^{\frac{3}{2}}$ gives the switching exponent to be $n_x = \frac{3}{2}$.

At $K_1^{\mathrm{eff}} > 0, K_2 = 0$, switching exponent is calculated to be 2, and at $K_1^{\mathrm{eff}} = 0, K_2 > 0$, the exponent is calculated to be $\frac{3}{2}$. Matsumoto *et al.* have calculated that the exponent of Eq. (S1) changes with the ratio K_2/K_1^{eff} of the magnetic anisotropy constants; $n_x = 2$ at $\frac{K_2}{K_1^{\mathrm{eff}}} = 0$, and n_x decreases with the increase of the ratio, and $n_x = \frac{3}{2}$ at $\frac{K_2}{K_1^{\mathrm{eff}}} = \frac{1}{4}$ [7]. Further increase of the ratio gives an undershoot of n_x down to 1.4, and gives the $n_x = \frac{3}{2}$ at $\frac{K_2}{K_1^{\mathrm{eff}}} = 0.5$. Our experimental result in the main text shows the transition of the exponent from 2 to $\frac{3}{2}$ between $0.43 < \frac{K_2}{K_1^{\mathrm{eff}}} < 0.45$. The discrepancy between experimental result and numerical calculation is most probably due to some realistic factors in the nanoscale magnetic tunnel junctions, *e.g.*, micromagnetic effects from the distribution of the stray field from the reference layer in the free layer, distribution of the demagnetising field, and the distribution of the magnetisation itself.

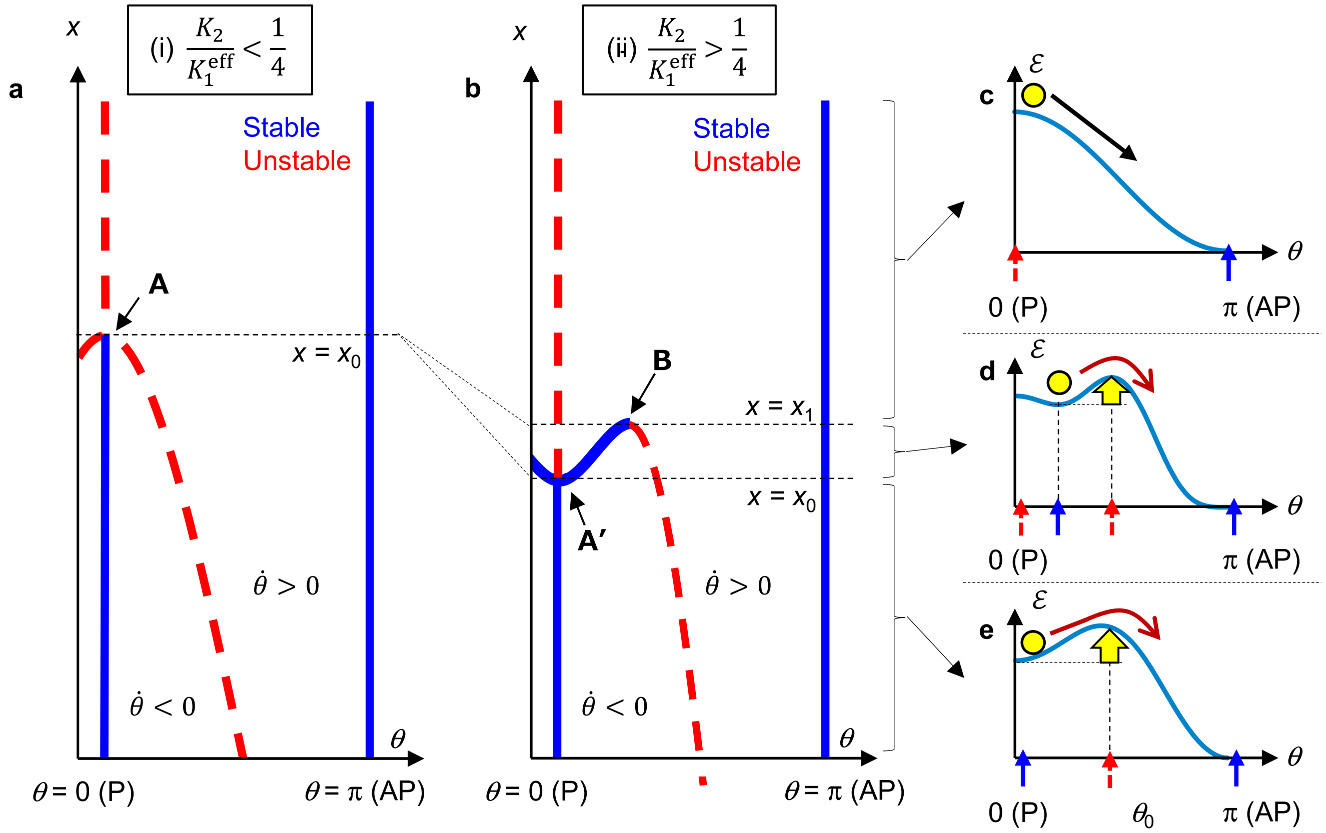

Fig. S3 | Types of the bifurcations and corresponding potential landscape for Landau-Lifshitz-Gilbert equation with uniaxial magnetic anisotropy and magnetic field. a,b, $\dot{\theta}$ as functions of external input x (field and/or current) and polar angle θ of magnetisation direction calculated by Eq. (S9) at the ratio K_2/K_1^{eff} of the first and second-order uniaxial anisotropy field constants **(a)** to be smaller than 1/4 and **(b)** to be larger than 1/4. **c-e**, potential landscape and the magnetisation dynamics for different external input.

References

1 Tulapurkar, A. A. *et al.* Spin-torque diode effect in magnetic tunnel junctions. *Nature* **438**, 339-342, doi:10.1038/nature04207 (2005).

2 Nozaki, T. *et al.* Electric-field-induced ferromagnetic resonance excitation in an ultrathin ferromagnetic metal layer. *Nat. Phys.* **8**, 491-496, doi:10.1038/nphys2298 (2012).

3 Kanai, S., Gajek, M., Worledge, D. C., Matsukura, F. & Ohno, H. Electric field-induced ferromagnetic resonance in a CoFeB/MgO magnetic tunnel junction under dc bias voltages. *Appl. Phys. Lett.* **105**, 242409, doi:10.1063/1.4904956 (2014).

4 Okada, A. *et al.* Electric-field effects on magnetic anisotropy and damping constant in Ta/CoFeB/MgO investigated by ferromagnetic resonance. *Appl. Phys. Lett.* **105**, 052415, doi:10.1063/1.4892824 (2014).

5 Okada, A., Kanai, S., Fukami, S., Sato, H. & Ohno, H. Electric-field effect on the easy cone angle of the easy-cone state in CoFeB/MgO investigated by ferromagnetic resonance. *Appl. Phys. Lett.* **112**, 172402, doi:10.1063/1.5026418 (2018).

6 Strogatz, S. H. *Nonlinear Dynamics and Chaos: With Applications to Physics, Biology, Chemistry, and Engineering (Studies in Nonlinearity)* (CRC Press, 2001).

7 Matsumoto, R., Arai, H., Yuasa, S. & Imamura, H. Efficiency of Spin-Transfer-Torque Switching and Thermal-Stability Factor in a Spin-Valve Nanopillar with First- and Second-Order Uniaxial Magnetic Anisotropies. *Phys. Rev. Appl.* **7**, 044005, doi:10.1103/PhysRevApplied.7.044005 (2017).
